# Resistance patterns and gene expression profiles of *Arcobacter butzleri* under exposure to selected antibiotics and disinfectants

**DOI:** 10.1016/j.crmicr.2026.100631

**Published:** 2026-06-08

**Authors:** Elisabetta Chiarini, Davide Buzzanca, Kurt Houf, Valentina Alessandria

**Affiliations:** a Department of Agricultural, Forest, and Food Science, University of Turin, Largo Paolo Braccini 2, Grugliasco, Torino, Italy; b Department of Veterinary and Biosciences, Faculty of Veterinary Medicine, Ghent University, Merelbeke, Belgium

**Keywords:** RNA-seq, AMR, One health, Virulence, Foodborne pathogen, Disinfectants

## Abstract

The rapid emergence and spread of antimicrobial resistance (AMR) pose a major global threat to public health. Beyond antibiotics, disinfectants used in food-processing environments may also exert selective pressure that promotes bacterial responses overlapping with antibiotic resistance mechanisms. This study explored the interconnection between antibiotic and disinfectant resistance of the pathogen *Arcobacter butzleri* by combining phenotypic and transcriptomic analyses. Thirty-one strains isolated from a poultry slaughterhouse were screened for susceptibility to antibiotics from different classes and to commonly used disinfectants. Following an initial screening, 3 representative strains were selected for in-depth analyses and exposed to erythromycin and amoxicillin – clavulanic acid, an amine-based and a sodium hypochlorite with caustic potash disinfectants. Bacterial load, metabolic activity, and transcriptomic responses, using RNA sequencing (RNA-seq), were assesed. All strains exhibited high levels of antibiotic resistance, including resistance to tetracycline and ampicillin. Antibiotic exposure was characterised by minimal reductions in bacterial load and few differentially expressed genes. In contrast, disinfectant exposure induced pronounced, strain-specific responses and resulted in the activation of multiple adaptive pathways. Differential gene expression involved chemotaxis regulators, DNA recombination, detoxification processes, Gram-negative cell wall components, protein and RNA processing systems. Notably, several genes associated with virulence and AMR (*tonB, hecB, mexA, mexB, mviN, phoP, tetR*, and *ylaC*), were overexpressed following disinfectants exposure, suggesting potential selection of virulence determinants following disinfectants exposure. The ability of *A. butzleri* to activate strain-specific adaptive mechanisms in response to antibiotics and disinfectants exposure may facilitate its persistence in food-processing environments and contribute to its pathogenic potential.

## Highlights

• Disinfectants trigger adaptive, strain-specific responses in *Arcobacter butzleri.*
• Overexpression of AMR and virulence genes in *A. butzleri.*
• Antibiotics induced limited transcriptomic changes.

## Graphical abstract

**Figure:**
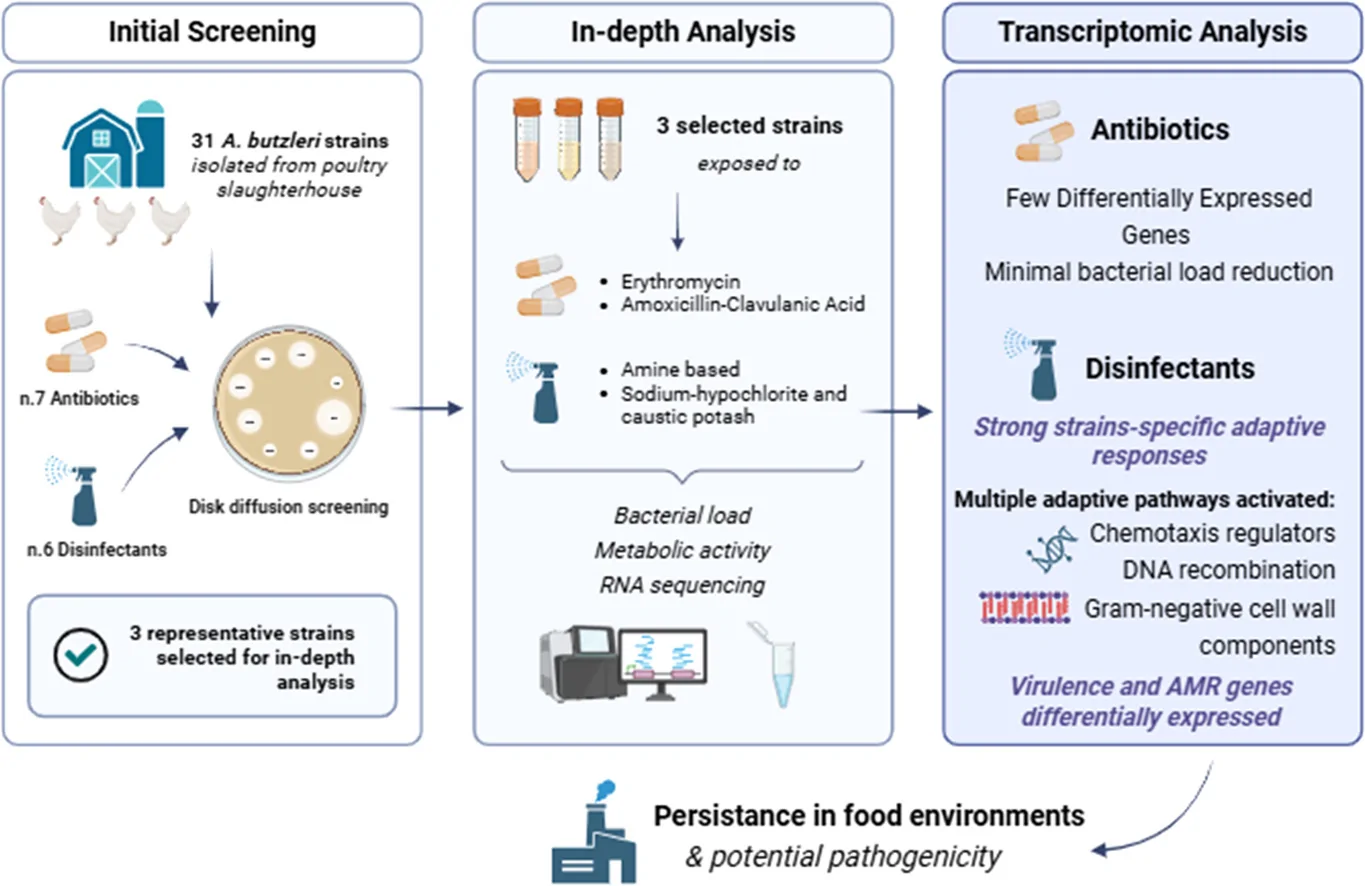

**Abbreviations**
Amoxicillin: AMO
Amoxicillin - clavulanic acid: AMC
Ampicillin sodium salt: AMP
analysis of variance: ANOVA
antibiotic: ATB
antimicrobial resistance: AMR
ATP-binding cassette: ABC
Ciprofloxacin: CIP
Clustered Regularly Interspaced Short Palindromic Repeats: CRISPR
coding DNA sequences: CDSs
colony forming unit: CFU
differentially expressed genes: DEGs
disinfectant: DIS
Erythromycin: ERY
European Committee on Antimicrobial Susceptibility Testing: EUCAST
false discovery rate: FDR
fold change: FC
Gentamicin: GEN
horizontal gene transfer: HGT
Kruskal-Wallis: K-W
multidrug resistance: MDR
3-(4,5-dimethylthiazol-2-yl)-2,5-diphenyltetrazolium bromide: MTT
National Center for Biotechnology Information: NCBI
optical density: OD
quaternary ammonium compounds: QACs
Resistance Nodulation Division: RND
RNA sequencing: RNA-seq
Tetracycline: TET
Turin University Culture Collection: TUCC
World Health Organisation: WHO

## 1 Introduction

Antimicrobial resistance (AMR) is one of the major public health challenges of the 21st century. This multifaceted phenomenon affects the treatment of human infections, animal health and the safety of agri-food chains. Addressing AMR requires an integrated approach, consistent with the One Health paradigm, which recognizes the interconnection of human, animal, and environmental health (World Health Organization, 2024). The emergence and spread of AMR are driven by the extensive use of antibiotics in human and veterinary medicine, agricultural practices, and the globalisation of food chains, underscoring the need for coordinated mitigation strategies (Chan, 2017; Murray et al., 2022; Velazquez-Meza et al., 2022). However, selective pressures contributing to AMR extend beyond therapeutic antimicrobials. Non-therapeutic antimicrobial agents, such as disinfectants, are widely used for cleaning and sanitising environments and surfaces to reduce microbial contamination (Rozman et al., 2021; Van Dijk et al., 2022). Disinfection practices play a key role at all stages of the food chain, particularly for surfaces, and equipment in direct contact with food. The choice of disinfectants is therefore critical (Maillard, 2018). Disinfectant selection must consider factors such as spectrum of activity, application conditions, and target microorganisms, as inappropriate or suboptimal use may promote the selection of resistant bacterial populations (Agüeria et al., 2021). Several parameters, including temperature, exposure time and disinfectant concentration, directly affect disinfection effectiveness (Rutala and Weber, 2024). Additionally, biofilm formation, water characteristics and intrinsic microbial resistance can further reduce disinfectant efficacy (Rozman et al., 2021; Tong et al., 2021). The most used disinfectants are based on quaternary ammonium compounds (QACs), chlorine derivatives, alcohols or hydrogen peroxide (Beier et al., 2017; Maillard, 2018).

Several studies have shown that exposure to disinfectants can induce adaptive bacterial responses that overlap with antibiotic resistance mechanisms, raising concerns about their potential contribution to the AMR landscape (Rozman et al., 2021; Van Dijk et al., 2022). Bacteria can develop intrinsic resistance through mechanisms such as altered membrane permeability, efflux systems, target site mutations, and enzymatic degradation (Novelli and Bolla, 2024). Among these, efflux systems are widespread and confer resistance to multiple antimicrobials (Chiarini et al., 2024; Gaurav et al., 2023). In addition, acquired resistance, mediated by horizontal gene transfer (HGT) of chromosomal and extra-chromosomal resistance determinants, represents a major challenge (Munita and Arias, 2016; Reygaert, 2018; Tong et al., 2021).

According to the World Health Organization (WHO), the priority bacterial pathogens in food chains include *Salmonella* spp., *Campylobacter* spp., enteropathogenic *Escherichia coli, Listeria monocytogenes, Staphylococcus aureus, Enterococcus* spp., *Klebsiella* spp. and *Pseudomonas aeruginosa.* All have been reported in association with environmental or food sources (WHO, 2024). Besides, emerging foodborne bacteria such as *Arcobacter butzleri,* are of particular concern due to their documented association with various diseases (Chiarini et al., 2025). *A. butzleri* is a Gram-negative bacterium belonging to the *Arcobacteraceae* family, and it has been identified as a zoonotic pathogen (Buzzanca et al., 2023; On et al., 2020). Since 1990, several infections caused by *A. butzleri* have been reported worldwide (Lappi et al., 2013; Vandamme et al., 1992). Gastrointestinal infections represent the most common manifestation across all age groups, although cases of bacteraemia have also been reported (Lappi et al., 2013; Ramees et al., 2017; Tang et al., 2023). *A. butzleri* has been isolated from a wide range of sources, including animals, humans and food products (Buzzanca et al., 2024; Chiarini et al., 2024). Strains isolated from animals and meat have shown multiple resistance against several classes of antibiotics (Chiarini et al., 2024; Gabucci et al., 2023; Isidro et al., 2020). Moreover, the bacterium is almost ubiquitous in food-processing environments, where it can form biofilms and adhere to various surfaces (Girbau et al., 2017). Although *A. butzleri* is not included in the WHO priority list, it is overlooked due to the frequent misidentification as *Campylobacter* spp., leading to its underestimation (Buzzanca et al., 2023; Kerkhof et al., 2021). Amid the escalating global challenge of antimicrobial resistance, there is a critical need for advanced analytical approaches capable not only of detecting resistance determinants but also of elucidating their transcriptional activity and regulatory dynamics (Darby et al., 2023).

This study aimed to use RNA sequencing (RNA-seq) to characterise strain-specific transcriptomic responses of *A. butzleri* strains from a single poultry slaughterhouse following exposure to antibiotics and disinfectants. Whole-genome analyses confirmed that the strains represent independent genetic distinctiveness (Chiarini et al., 2024), enabling controlled comparison of regulatory responses under defined exposure conditions. Sampling from the same facility provided environmental consistency, minimizing extrinsic variation and enabling controlled comparison of transcriptional responses, in line with established RNA-seq experimental design principles that emphasize reducing non-biological variability (Gonzalez-Torres et al., 2015; Kimes et al., 2014). By integrating RNA-seq with antimicrobial challenge assays, this work provides high-resolution insights into transcriptional adaptations to antimicrobial stress of *A. butzleri*, extending beyond previous phenotypic resistance assessments.

## 2 Materials and methods

### 2.1 Strains and antimicrobial resistance agar disk diffusion method

Thirty-one *A. butzleri* strains (**Table S1**) isolated from a chicken slaughterhouse (Chiarini et al., 2024), deposited in the Turin University Culture Collection (TUCC) (https://www.tucc.unito.it/it), together with the *A. butzleri* type strain LMG10828^T^, were evaluated for the susceptibility test with the disk diffusion method. Detailed information on their origin and genomic characterisation has been reported previously (**Table S1**) (Botta et al., 2024; Chiarini et al., 2024). Six commercially available disinfectants (Table 1), commonly used for cleaning and sanitisation in food processing plants, were selected to represent distinct antimicrobial mechanisms. The concentrations used were based on those specified in the product data sheets using an intermediate value compared to the recommended percentage concentration range (Table 1). The antibiotics tested were gentamicin (G1264–5 GR, Sigma-Aldrich, Milan, Italy), erythromycin (CT0020B, Thermo Scientific, Milan, Italy), ampicillin sodium salt (A9518–5 G, Sigma-Aldrich), amoxicillin (A8523–5 G, Sigma-Aldrich), amoxicillin - clavulanic acid (SMB00607–1, Sigma-Aldrich), tetracycline (CT0054B, Thermo Scientific) and ciprofloxacin (CT0425B, Thermo Scientific), the concentrations used are listed on the Table 1. In this study, antibiotic resistance of the strains was reassessed using the disk diffusion method to serve as a baseline for comparison with disinfectant susceptibility, providing an integrated view of antimicrobial responses before transcriptomic analysis.

**Table 1:** Antimicrobial compounds tested. List of disinfectants and antibiotics used for the susceptibility testing. The disinfectants marked with an “*” are those used in the slaughterhouse of origin of the strains. In the case of disinfectants D and F (mean usage concentration) and antibiotics AMC and ERY (EUCAST MIC breakpoints), the concentrations and exposure times (minutes) used in the cell layer exposure test are also indicated.

**Disinfectant**	**Description**	**Concentration tested (v/v) for the disk diffusion method**	**Time of exposure and concentration tested (v/v)**
A	Concentrated liquid descaling agent based on peracetic acid with foaming action	3%	-
B*	Liquid disinfectant based on peracetic acid and hydrogen peroxide	0.50%	-
C	Liquid disinfectant based on quaternary salts of ammonium	0.60%	-
D	Concentrated amine-based liquid disinfectant	1.25%	15 min-0.5%
E	Alkaline foaming disinfectant based on sodium hypochlorite with caustic soda	9.50%	-
F*	Alkaline foaming disinfectant based on sodium hypochlorite with caustic potash	8.5%	10 min-2%
**Antibiotic**	**Structural Classes**	**Disk content (µg)**	**Time of exposure and concentration tested (mg/L)**
Ciprofloxacin - CIP	Floroquinolones	5	-
Tetracycline - TET	Tetracyclines	30	-
Amoxicillin - clavulanic acid - AMC	Penicillin	30	60 min-8 mg/L
Amoxicillin - AMO	Penicillin	25	-
Ampicillin sodium salt - AMP	Penicillin	25	-
Erythromycin - ERY	Macrolide	15	60 min-8 mg/L
Gentamicin - GEN	Aminoglycoside	10	-

The protocol of the European Committee on Antimicrobial Susceptibility Testing (EUCAST breakpoint tables, v. 14.0, www.eucast.org) was used for antibiotic susceptibility tests, and for the disinfectant susceptibility tests with some modifications. Values for *Campylobacter jejuni* and *Campylobacter coli* were used to determine the resistance and susceptibility of *A. butzleri*. Where these values were lacking, reference was made to the values established for Enterobacteriales.

The bacterial suspension was prepared as follows. One single fresh colony of each strain was transferred into 3 mL of *Arcobacter* broth (CM0965, Oxoid, Milan, Italy) and incubated for 48 h in microaerobic conditions (Oxoid CampyGen 3.5 L Sachet, CN0035A) at 30 °C. One hundred microliters of the bacterial suspension were then transferred into 3 mL of *Arcobacter* broth and then incubated for 18 h at 30 °C. The concentration of the grown broth culture was set to 0.5 McFarland (Remel, Thermo Fisher Scientific). Then, 150 µL of each bacterial suspension was inoculated onto Müeller-Hinton agar medium (CM0337B, Oxoid). In the case of antibiotics, 5% defibrinated horse blood and 20 mg/mL β-NAD were added to Müeller-Hinton agar medium. Sterile discs (Macherey-Nagel, Germany, 484,000) were placed on each plate inoculated with each antimicrobial compound at the chosen test concentration (Table 1). Plates were incubated for 48 h at 30 °C. Disinfectant assays were conducted under aerobic conditions to mimic real-world environmental exposure. Antibiotic susceptibility testing was carried out under microaerobic conditions, as required by EUCAST guidelines for *Campylobacter* spp. The inhibition halo was measured at the end of the incubation period. Each compound was tested in technical double replicates and biological triplicate.

### 2.2 Resistance assessment following exposure, microbial load and MTT staining assay

Two disinfectants with distinct active ingredients but similar inhibition profiles, and two antibiotics representing different mechanisms of action, were selected for further analysis in three *A. butzleri* strains from the original 31 considering the disk diffusion results. The *A. butzleri* strains selected were TUCC00000927 (BZe322), TUCC00000928 (BZe327) and TUCC00000930 (BZe363). An individual *A. butzleri* colony was transferred to 3 mL of *Arcobacter* broth and incubated for 48 h at 30 °C, under microaerobic conditions. For the assay, Müeller-Hinton agar medium (CM0337B, Oxoid) was inoculated with 500 μL of bacterial suspension. The plates were then incubated for 48 h at 30 °C under microaerobic conditions. The bacterial cell layers were incubated in Ringer’s solution under both control and treatment conditions, with antibiotic or disinfectant compounds added only to the treated samples, ensuring that all comparisons were performed against the same Ringer-based baseline. Two millilitres of solutions containing disinfectants (disinfectant F, 2% v/v; disinfectant D, 0.5% v/v) or antibiotics (erythromycin, 8 mg/L; amoxicillin – clavulanic acid, 8 mg/L) were added to the formed bacterial layer in the Petri dishes (63.6 cm^2^). Disinfectant F was applied for 10 min, disinfectant D for 15 min, and both antibiotics were applied for 60 min each (Table 1). Ringer's solution (Neogen, U.S.A, Michigan, Lansing, NCM0191K) was used as the blank control, containing no disinfectants or antibiotics. The plates were then incubated at 25 °C. After the predetermined incubation time, the cell layer was collected and centrifuged twice at 10,000 x g for 7 min. The bacteria cells were then resuspended in 500 µL of Ringer's solution to perform serial decimal counts evaluating the bacterial load (Log_10_ CFU/mL). The differences in bacterial load between the treated and control samples were assessed using the following formula: Δ Log_10_ CFU/mL = Log_10_ CFU/mL _test_ (bacterial load of strains exposed to antimicrobial) – Log_10_ CFU/mL _control_ (bacterial load of strains exposed to Ringer’s solution).

The bacterial metabolic activity was evaluated for 100 µL of bacterial suspension after initial optical density (OD) measurement (Bioteck; Synergy HT) at 630 nm. Ten microliters of 3-(4,5-dimethylthiazol-2-yl)-2,5-diphenyltetrazolium bromide (MTT) (Apollo scientific, Manchester, UK; BID2165) were then added. The samples were vortexed, incubated for 20 min at 37 °C in the dark and then centrifuged at 10,000 x g for 1 min. The pellet was resuspended in 100 µL of PBS 1X and 100 µL of isopropanol (VWR, Radnor, PA, USA, 60,482). The OD of this suspension was measured at 490 nm. The metabolic activity of the samples was analysed comparing the 490/630 ratios of the test and control samples. The MTT assay was performed in technical double replicates and biological triplicate.

### 2.3 RNA extraction and sequencing

RNA extraction was performed using the RNeasy Mini Kit (Qiagen; 74104) according to the manufacturer's instructions. Samples were prepared from 500 µL of the bacterial suspensions exposed to disinfectants and antibiotics as described above adding 1 mL of RNAprotect Bacteria Reagent (Qiagen; 76506). The samples were incubated at 25 °C for 5 min, then centrifuged for 10 min at 5000 x g. The bacterial cells were resuspended in 100 μL of TE buffer (30 mM Tris–HCl and 1 mM EDTA pH 8.0) containing 15 mg/mL of lysozyme (Sigma-Aldrich; 62971) and 50 mg/mL of proteinase K (APOSBIP4205, Apollo Scientific; Manchester, U.K.). The samples were mixed and incubated at room temperature for 10 min at 600 rpm (Thermomixer compact, Eppendorf; Hamburg, Germany). After the addition of 350 μL of Buffer RLT, the RNA extraction was performed according to the manufacturer's protocol of the RNeasy Mini Kit (Qiagen; 74104). The nucleic acids, eluted in 35 µL of the elution buffer, were subjected to DNA digestion by treatment with DNAse buffer 10X (AM2222, Ambion, Thermo Fisher Scientific UAB, Vilnius, LT) and DNAse I (2 U). (AM2222, Ambion, Thermo Fisher Scientific UAB, Vilnius, LT). The samples were then incubated for 2 h at 37 °C at 600 rpm (Thermomixer compact, Eppendorf). A further hour of incubation at 37 °C, 600 rpm (Thermomixer compact, Eppendorf) was performed after the addition of 1.5 µL of DNAse I. Finally, the enzyme was inactivated by the addition of 5 µL of 0.5 mM EDTA, pH 8.0 (Sigma-Aldrich, Milan, Italy), followed by 10 min of incubation at 75 °C (Thermomixer compact, Eppendorf). The amount and quality of RNA were evaluated with Nanodrop (Thermo Fisher; Waltham, Massachusetts, U.S.A; ND-1000), and with an electrophoretic run in agarose gel 0.8% (NIPPON Genetics; AG02) in TBE 1X for 30 min at 120 V.

The RNA-seq library preparation and sequencing were performed by Novogene company (Munich, Germany). After assessing RNA quality using a bioanalyzer, ribosomal RNA was removed, followed by ethanol precipitation. The first strand cDNA was synthesized using random hexamer primers after fragmentation. During the second strand cDNA synthesis, dUTPs were replaced with dTTPs in the reaction buffer. After end repair, A-tailing, adapter ligation, size selection, enzyme digestion, amplification, and purification the directional library was ready. Library quantification was performed with Qubit and real-time PCR, while size distribution evaluation with bioanalyzer. Quantified libraries were pooled and sequenced on Illumina NovaseqX platforms (3 Gb per sample; 150 bp; PE).

### 2.4 Bioinformatics analysis

Unless otherwise stated, the following bioinformatics tools were used with default options. The *A. butzleri* genomes of three strains (BZe322, BZe327 and BZe363; NCBI accession PRJNA986324) destined for transcriptomic study (Chiarini et al., 2024) were functionally annotated with Prokka 1.14.6 (Seemann, 2014). The raw sequence reads, delivered as adapter-free clean reads by the sequencing provider (Novogene company), were further quality-trimmed using AdapterRemoval v2.3.2 (–minquality 30, –trimns, –maxns 10, –trim5p 2, –trim3p 2) (Schubert et al., 2016). The reads quality check was performed with FastQC v0.12.1 (https://www.bioinformatics.babraham.ac.uk/projects/fastqc/; link opened on 12/30/2025) and subsequently aligned to Prokka v1.14.6 predicted coding DNA sequences (CDSs; .ffn files) with Bowtie2 v2.4.0 to obtain read counts corresponding to CDSs (Langmead and Salzberg, 2013). SUPER-FOCUS 1.4.1 (-db DB_98) was used to obtain information on read pathways abundances.

The gene sequence identified as hypothetical proteins was aligned, searching for homologous genes, using BlastP (https://blast.ncbi.nlm.nih.gov/Blast.cgi?PAGE=Proteins; link opened on 06/15/2025) and SWISS-MODEL (https://swissmodel.expasy.org/; link opened on 06/15/2025) (Altschul et al., 1997; Waterhouse et al., 2018). The DEGs analysis was represented graphically using a Venn diagram (Heberle et al., 2015).

### 2.5 Statistical analysis

Statistical analysis was performed using RStudio software version 4.2.2 (2022–10–31 ucrt). Shapiro-Wilk and Levene's W-tests were used to check the normality and homogeneity of the data, respectively. The Kruskal-Wallis (K-W) test and analysis of variance (ANOVA) were used to assess overall differences and variations between multiple groups. These tests were used for non-parametric (K-W) and parametric (ANOVA) data. Dunn's test with Bonferroni’s correction was used in the post-hoc analyses for non-parametric data unless differentially indicated, while Tukey's test was used for parametric data. EdgeR v4.4.2 (Robinson et al., 2010) and deseq2 v1.46.0 (Love et al., 2014) were used to identify differentially expressed genes (DEGs) between conditions. Genes were considered differentially expressed when the log2 fold change (log_2_ FC) was greater than 1 or less than −1, and the false discovery rate (FDR) adjusted p-value was below 0.05, unless otherwise specified.

## 3 Results and discussion

### 3.1 Antibiotics and disinfectants resistance of *A. butzleri* strains

**Fig. 1:**
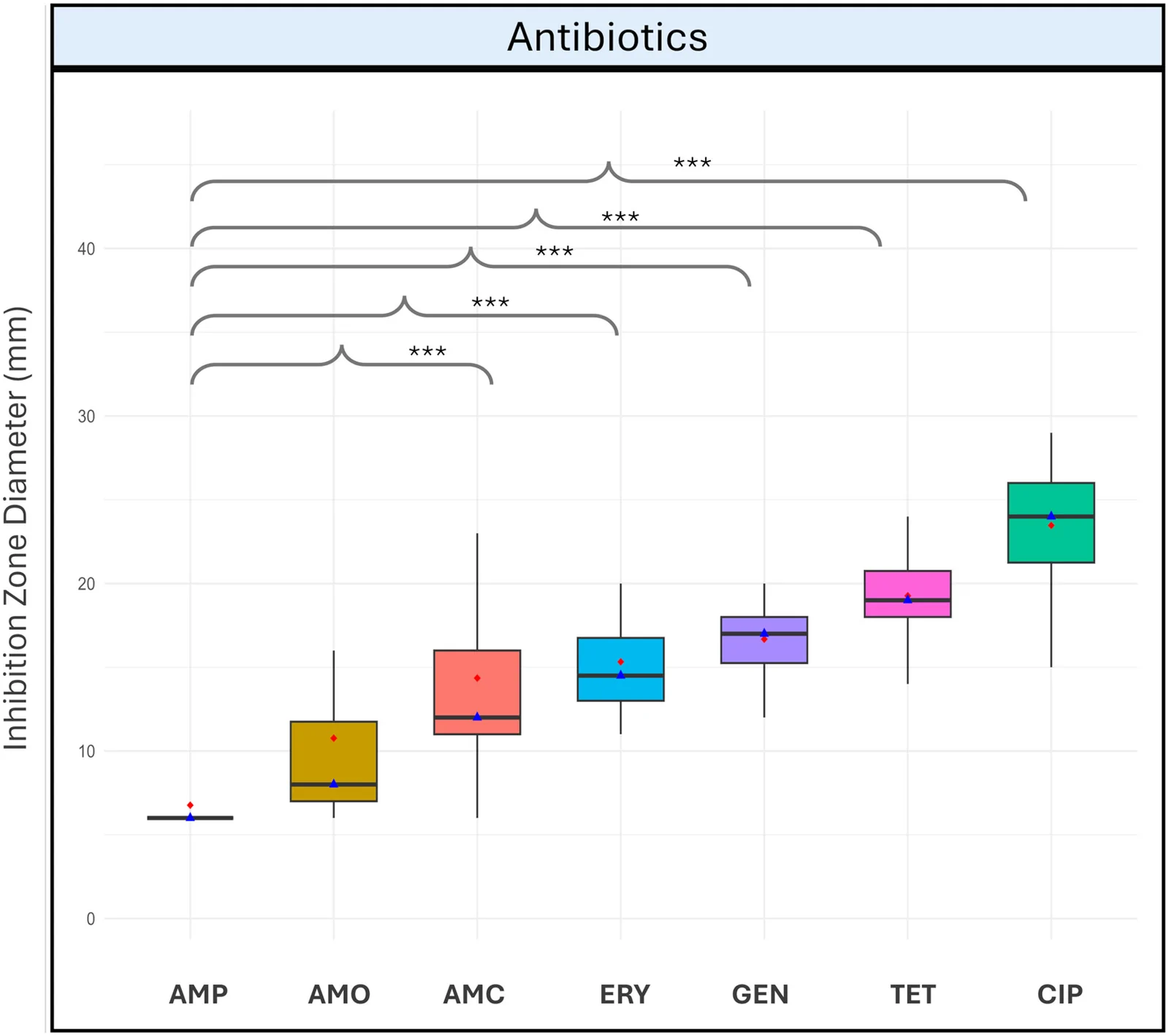
**Boxplot showing the inhibition zone diameter (mm) produced by tested antibiotics** (Table 1). A blue triangle indicates the median, and the mean is marked by a red diamond. Statistical significance between groups was assessed using Kruskal–Wallis followed by Dunn’s post hoc test (*p*-value < 0.05). Asterisks (***) indicate the significance level (*p-*value < 0.001).

Thirty-one *A. butzleri* strains analysed in this study were originally isolated from poultry slaughterhouse equipment and chicken carcasses (**Table S1**). Two of the disinfectants tested were used in rotation for cleaning and sanitising surfaces and equipment in the slaughterhouse (B and F, Table 1). As the strains were collected after exposure to disinfectants, they may already carry adaptive traits; nevertheless, this study aims to evaluate their resistance and related mechanisms under controlled in vitro conditions. No significant differences in resistance were observed considering strains from carcasses and equipment within the same facility (ANOVA/Tukey's test, *p*-value > 0.05) (**Table S1**), whereas differences were detected depending on the type of disinfectant used (K-W/Dunn’s test, *p*-value < 0.05) (Fig. 1, **Figure S1; Table S2-S3**). The lack of significant resistance variation between strains from chicken carcasses and equipment within the same slaughterhouse suggests that resistance is not associated with the specific isolation site. This may reflect cross-contamination within the slaughterhouse (Botta et al., 2024; Chiarini et al., 2024). Although the disinfectant concentrations used in this study reflect recommended operational levels, it is likely that bacteria in food-processing environments may be exposed to residual sub-lethal concentrations on surfaces following sanitation procedures. These conditions may contribute to the selection of adaptive transcriptional responses observed in this study and are relevant to post-sanitation environments where residual disinfectant levels may exert selective pressure on surviving bacteria and contribute to adaptive responses observed in vitro. Disinfectants A and B, were both based on peracetic acid and B additionally containing hydrogen peroxide, produced the largest inhibition zones across all strains (K-W/Dunn’s test, *p*-value < 0.05), with mean diameters of 35.42 ± 2.38 mm and 31.18 ± 3.22 mm (**Figure S1, Table S2**). The other disinfectants produced smaller inhibition zones, mostly below 15 mm. The smallest zones were observed for disinfectants E and F, both alkaline foaming agents based on sodium hypochlorite and containing caustic soda (E) or caustic potash (F), with mean diameters of 12.28 ± 2.76 mm and 11.39 ± 0.83 mm, respectively (**Figure S1, Table S2**). Among the antibiotics tested (Fig. 1), ciprofloxacin showed the largest inhibition zone (average 23.47 ± 4.63 mm), followed by tetracycline (average 19.26 ± 2.34 mm) (K-W/Dunn’s test, *p*-value < 0.05). In contrast, the smaller inhibition zones were recorded for ampicillin (average 6.76 ± 0.67 mm), and for amoxicillin (average 10.76 ± 5.35 mm) (K-W/Dunn’s test, *p*-value < 0.05). Inhibition zones produced by ampicillin were significantly smaller than those of all other antibiotics tested (K–W/Dunn’s test, *p*-value < 0.001), except amoxicillin (*p*-value < 0.01) (Fig. 1, **Table S2-S3**). EUCAST guidelines were used to interpret the antibiotic inhibition zone diameters as resistant (R), increased resistance (I) or susceptible (S) phenotypes (**Table S3**). All *A. butzleri* strains tested were resistant to tetracycline and ampicillin. Among the strains, 93.75% were resistant to erythromycin, 81.25% to amoxicillin, and 78.12% to amoxicillin with clavulanic acid. In contrast, 31.25% of the strains showed intermediate resistance to ciprofloxacin, and 68.75% were susceptible to gentamicin (**Table S3**). Strains BZe306, BZe344, BZe363, BZg278, BZs158, BZs170, BZs252, BZs99 and the type strain LMG10828^T^ showed resistance against all antibiotic tested. Currently, no official antibiotic susceptibility breakpoints have been established for the genus *Arcobacter* spp., thereby limiting the precise interpretation of its antimicrobial resistance profiles (Buzzanca et al., 2024). Furthermore, the utilisation of surrogate breakpoints derived from related taxa (*Campylobacter* spp. and Enterobacterales) has the potential to influence the classification of isolates as susceptible or resistant. Consequently, the results of this study should be interpreted with the requisite degree of caution. Finally, no official or standardised breakpoint exists for defining disinfectant resistance (Maillard, 2018). Therefore, the disk diffusion assay used in this study should be considered as a preliminary screening approach aimed at comparing the relative activity of the tested disinfectants and highlighting potential differences among the investigated strains, rather than providing a definitive classification of disinfectant resistance.

### 3.2 Correlations between antibiotic resistances

Pearson’s correlation (**Figure S2, Table S5**) revealed the strongest positive correlation between AMO and AMC resistances, suggesting that clavulanic acid is ineffective against part of *A. butzleri* amoxicillin-resistant strains. A positive correlation was also observed between resistance to ERY and AMC. Moreover, the positive correlation between resistance to antibiotics with different mechanisms of action suggests that antimicrobial resistance genes may be associated with non-specific resistance strategies (Reygaert, 2018). This correlation underscores the phenomenon of cross-resistance among antibiotics belonging to different classes, each exhibiting a distinct mechanism of action and targeting distinct sites on the bacterial cell. Several underlying mechanisms may explain this observation, including the co-occurrence of resistance determinants, which may be co-localized on mobile genetic elements (Buzzanca et al., 2024).

### 3.3 Evaluation of microbial load and metabolic activity of strains after antimicrobial exposure

**Fig. 2:**
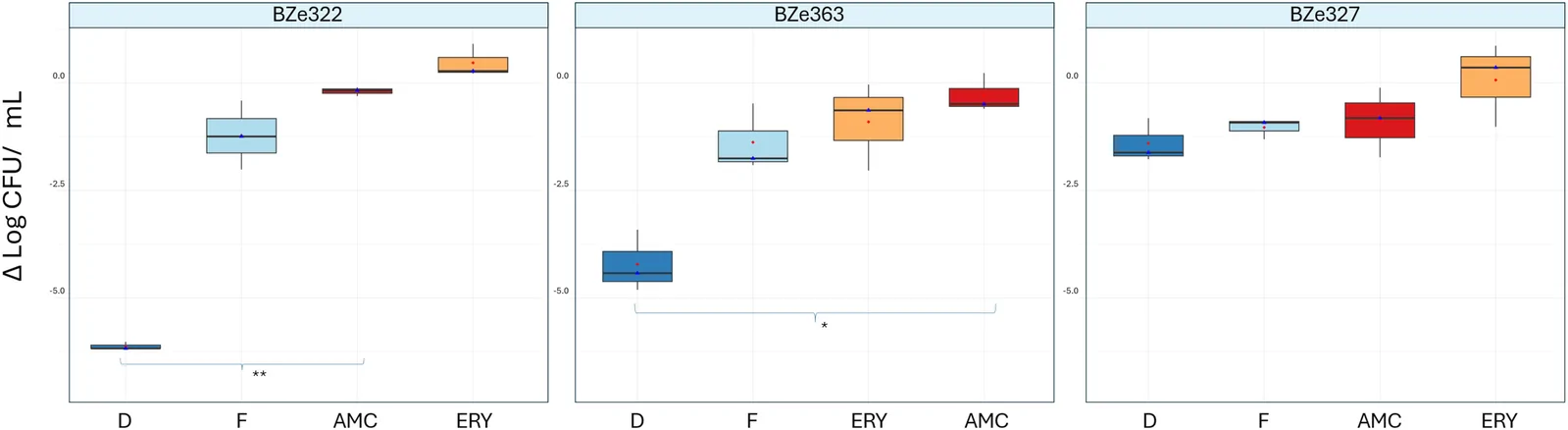
**Strains resistant load after exposure at antibiotics and disinfectants.** The boxplots show the results of the microbiological loads expressed as Δ Log_10_ CFU/mL. The median is indicated by a blue triangle, and a red diamond marks the mean. Statistical significance between groups was assessed using K-W followed by Dunn’s post hoc test (* = *p-*value < 0.05; ** = *p-*value < 0.01) (**Table S7**).

After evaluating antimicrobial susceptibility, strains BZe322, BZe327, and BZe363 were selected for transcriptomic analysis, based on their distinct AMR profiles (Fig. 2) as well as their genetic diversity, which was confirmed in a previous study (Chiarini et al., 2024). Strains were exposed to antibiotics for 1 hour and disinfectants for 10–15 min, consistent with recommended exposure times to induce biologically meaningful transcriptomic changes without causing excessive cellular stress (Merchel Piovesan Pereira et al., 2020; Poonawala et al., 2024). Results, expressed as the mean and standard deviation of the biological triplicate are shown in Fig. 2 and **table S6**. Bacterial load values (Δ Log_10_ CFU/mL) closer to zero indicate minimal reduction in bacterial load compared to the control condition.

Upon exposure to ERY, BZe322 and BZe327, showed positive Δ Log_10_ CFU/mL values of 0.5 ± 0.4 and 0.1 ± 1.0, respectively (**Table S6**), indicating that ERY did not reduce bacterial load compared to the control. Exposure to AMC, caused minimal reduction in all three strains (mean value - 0.5 ± 0.4) (Fig. 2). In contrast, disinfectant D (amine-based) reduced microbial loads for all three strains (mean value: −3.9 ± 0.4). The effect was most pronounced in strains BZe322 and BZe363, with Δ Log_10_ CFU/mL values of −6.1 ± 0.1 and −4.2 ± 0.7, respectively (K-W/Dunn’s test, *p-*value < 0.01) (Fig. 2, **Table S6**). Disinfectant F (caustic potash-based), showed low efficacy, reducing bacterial loads by −1.40 ± 0.8, −1.20 ± 0.8, and −1.0 ± 0.2, in strains BZe363, BZe322, and BZe327, respectively. This low activity may explain the survival of these strains on slaughterhouse surfaces and their subsequent isolation after routine disinfection (Chiarini et al., 2024).

The analysis focused on microbial load and cell viability to assess whether antimicrobial treatments affect viability relative to the control condition. Significant differences were observed between strains treated with antibiotics and those treated with disinfectants, using the MTT assay to assess metabolic activity (ANOVA/Tukey’s test, *p-*value < 0.05) (**Table S7**). BZe327 and BZe363, exposed to antibiotics, demonstrated higher cell viability compared to BZe322 under the same conditions (**Table S7**). Conversely, strain BZe322 exhibited the lowest cell viability when exposed to disinfectants compared to the other two strains (**Figure S3**). These strain-specific differences suggest the involvement of distinct adaptive and resistance mechanisms. The high genomic plasticity of *A. butzleri*, previously reported (Buzzanca et al., 2021; Chiarini et al., 2024), likely underlies these strain-specific responses.

### 3.4 Gene transcription analysis in the presence of antibiotics compared to controls

Transcriptomic profiles of antibiotic-treated samples were compared with those from untreated controls to elucidate bacterial response mechanisms following antibiotic exposure. The analysis revealed strain-specific transcriptional response after one hour of antibiotic exposure. BZe322 and BZe363 showed few or no DEGs compared to the control condition (log_2_ FC < −1; *p-*value [FDR] < 0.05; the lower log_2_ FC between DESeq2 and edgeR is reported in the text) (**Table S8**). Instead, strain BZe327 exhibited ten DEGs (log_2_ FC > 1 or < −1; *p-*value [FDR] < 0.05) in response to ERY exposure (**Table S8**). Furthermore, exposure to ERY in strain BZe327 increased bacterial growth, and general exposure to antibiotics increased cell viability (Fig. 2, **S2**).

**Fig. 3:**
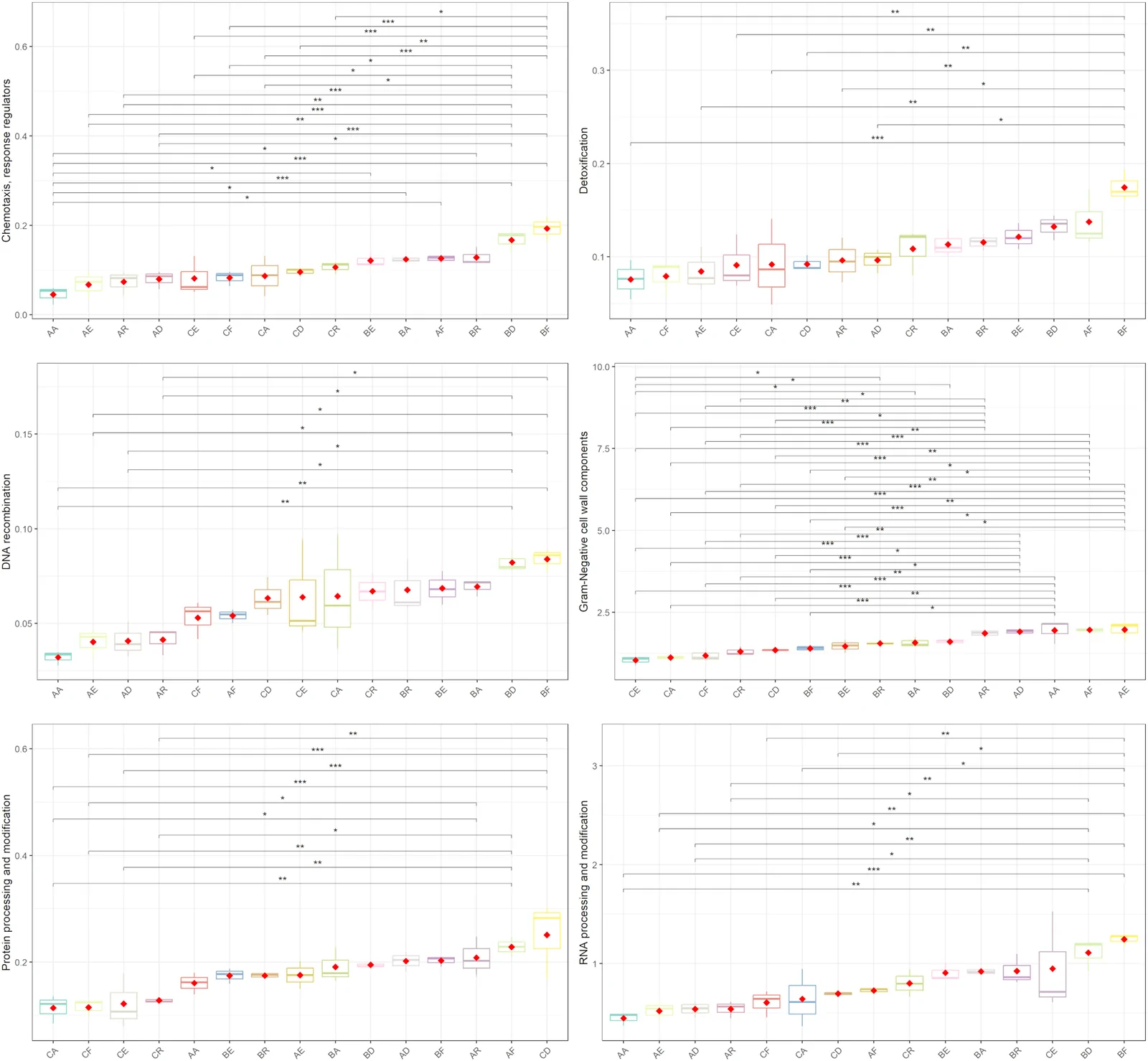
The box plots show the percentage reads from RNA-seq analysis related to several gene classes. The strains are indicated by letters A (BZe322), B (BZe363) and C (BZe327). The antimicrobial exposures are indicated by A (amoxicillin - clavulanic acid), E (erythromycin), F and D (disinfectant F and D) and R (control). Asterisks indicate significance levels: *** *p-*value *< 0.001*, **p-value *< 0.01*, *p-value *<0.05* (**Table S11**).

Functional classification of expressed genes and pairwise comparisons among strains under identical exposure conditions were subsequently performed (Fig. 3, **Table S11**). Reads belonging to the chemotaxis and response regulator gene classes were more abundant in strain BZe363 than in strain BZe322 under AMC (*p-*value < 0.05). The strain BZe322 demonstrated a higher percentage of reads associated with Gram-negative cell wall components under AMC exposure compared with BZe327, and under ERY exposure compared with strain BZe363 (*p-*value < 0.05) (Fig. 3). Furthermore, following exposure to antibiotics, DEGs mainly belonged to functional categories involved in ribosome biogenesis and the promotion of increased protein synthesis, as well as RNA modification and transcriptional regulation. These results are consistent with those from MTT staining (**Figure S3**), in which higher cell viability in strain BZe327 correlates with the expression of genes linked to ribosomal activity. Specifically, ribosomal proteins 50S, L27, and L1, were overexpressed during ERY exposure (**Table S8**). In *E. coli*, 50S ribosomal protein L27 plays a key role in assembling and maintaining the ribosomal large subunit, essential for bacterial growth and survival (Nevskaya et al., 2005; Wower et al., 1998). The overexpression of these genes is particularly relevant, given that erythromycin targets the 50S subunit of bacterial ribosomes, thereby inhibiting protein synthesis and ultimately impairing bacterial growth and replication (Krawczyk et al., 2024). Overexpression of the enzyme tRNA dimethylallytransferase (**Table S8**), involved in the final transcription regulation (Fleming et al., 2022), was observed after the exposure to ERY. It has been observed that one of the primary changes in macrolide antibiotic resistance was associated with RNA mutation, as well as methylation (Reygaert, 2018). Downregulated genes after ERY exposure included *ubiB*, an atypical kinase associated with coenzyme Q biosynthesis; EAL domain-containing protein with phosphodiesterase activity, the transcription factor *ypdB*, and the enzymes phytoene desaturase and deoxyribodipyrimidine photo lyase, which participate in DNA repair (**Table S8**). Phytoene desaturase plays multiple biological roles, including protection against oxidative stress and stabilisation of cell membranes (Liu et al., 2012). The impact of undersaturation on membrane fluidity and stability could be a consequence of the stress conditions to which it was subjected (Liu et al., 2012). Simultaneous downregulation of these genes suggests suppression of the stress response, DNA repair, and metabolic reprogramming. No genes were differentially expressed comparing AMC exposure to the control condition (*p*-value *> 0.05*). This lack of differential expression may be due to clavulanic acid, which inhibits enzymes involved in bacterial cell wall synthesis, thereby enhancing antimicrobial activity (Drawz and Bonomo, 2010).

### 3.5 Gene transcriptional analysis in the presence of disinfectants compared to the controls

**Fig. 4:**
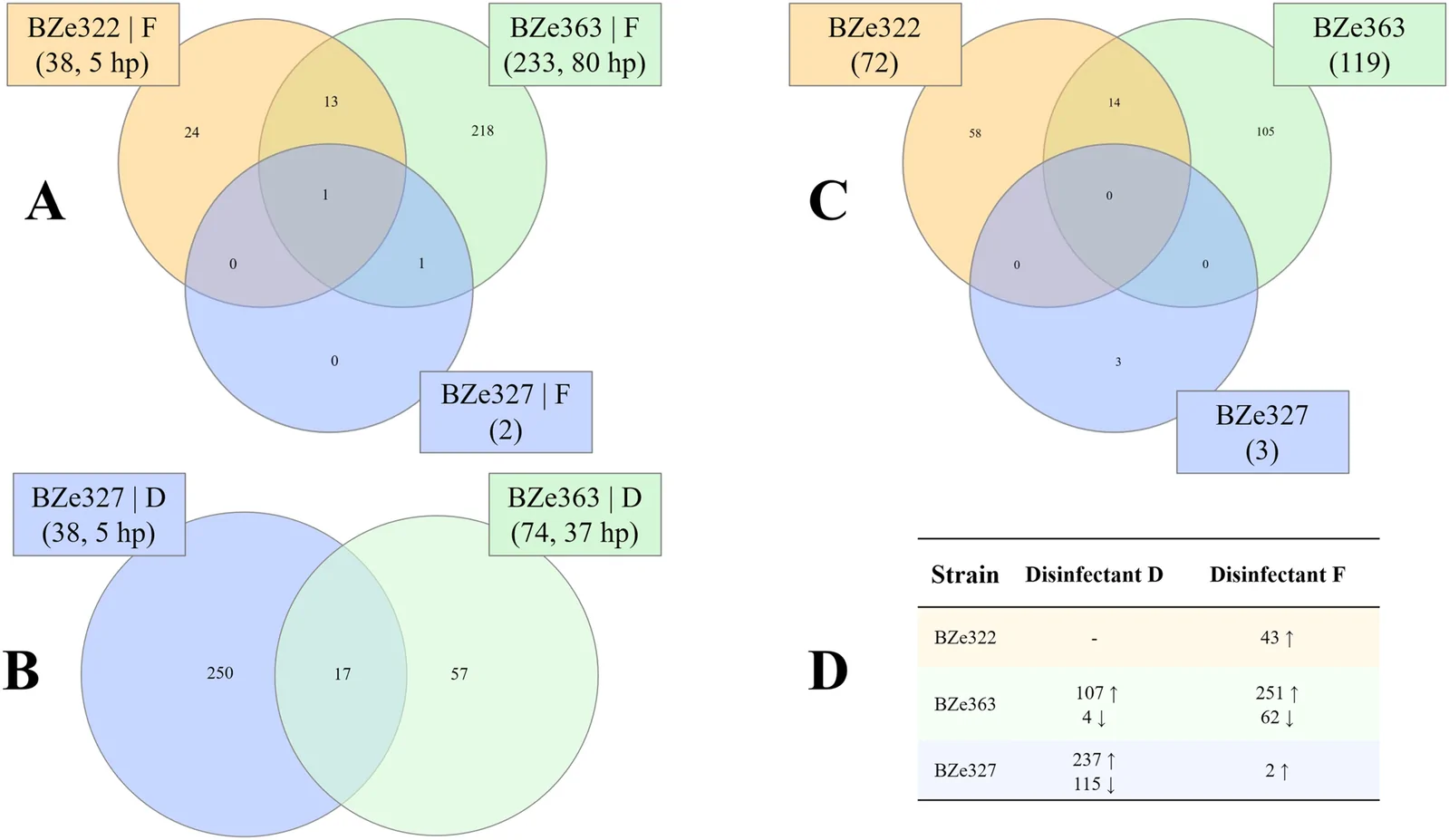
Venn diagram of DEGs (*p-value* [FDR] < 0.05) (**Table S9, S10**). The total number of DEGs is indicated in each respective square divided by strain. Hypothetical proteins (hp) have been indicated in brackets. **A)** Analysis of DEGs expressed after the treatment with disinfectant F; **B)** DEGs after treatment with disinfectant D; **C)** Genes differentially expressed in comparison between transcripts resulting from treatment with antibiotics and with disinfectants. Hypothetical proteins were excluded from the analysis; **D)** Summary tables of DEGs in three strains following disinfectant exposure, divided into overexpressed (↑) and under-expressed genes (↓) (log_2_ FC > 1 or < −1; *p-value* [FDR] < 0.05).

Differential gene expression was evaluated by comparing transcriptomes of disinfectant-exposed samples with untreated controls (**Figure S4**). Marked strain-specific differences were again observed in response to disinfectants exposure. In strain BZe322, 43 DEGs were overexpressed following exposure to disinfectant F (sodium hypochlorite-based with caustic potash). The exposure of BZe363 to disinfectant F led to the overexpression of 251 genes and the under-expression of 62 genes. Following exposure to disinfectant D (amine-based disinfectant), 107 genes were overexpressed and 4 genes were downregulated in BZe363. Strain BZe327 showed a contrasting response, with the strongest transcriptional changes observed after exposure to D. Of the 352 DEGs identified, 237 were overexpressed following disinfectant D treatment, whereas only two DEGs were detected after exposure to F (Fig. 4A-B, **S3, Table S8, S9**).

The disinfectants tested exhibited two distinct mechanisms of action. Active chlorine oxidises essential cellular components, including proteins and nucleic acids (Disinfectant F) (Fukuzaki, 2006). The amine-based disinfectant (D) acts as a cationic surfactant interacting with the cell membrane and consequently altering its permeability (Krawczyk et al., 2024). In response to disinfectant-induced stress, the most affected gene classes were related to chemotaxis response regulators, DNA recombination, detoxification, Gram-negative cell wall components, protein processing and modification, RNA processing and modification (Fig. 3, **Table S8**). These include genes associated with virulence and antimicrobial resistance, including *tonB, mexA, mexB, phoP, tetR, hecB* (*shlB*) and *mviN* (*murJ*) (Table 2) (Buzzanca et al., 2021; Buzzanca et al., 2023; Chiarini et al., 2024). Reads associated with genes belonging to the chemotaxis and response regulator classes demonstrated higher abundances in BZe363 than in BZe327, following exposure to disinfectant F (*p-*value < 0.05) (Fig. 3). Similarly, a high number of reads were observed in BZe363 compared with BZe322 following treatment with disinfectant D. Similar trends were observed for gene classes involved in DNA recombination and RNA modification (BZe363 > BZe322 under D) and detoxification (BZe363 > BZe327 under F) (Fig. 3). However, the gene class involved in protein processing and modification showed the highest read abundance in BZe322 following exposure to disinfectant F, rather than in BZe327 (Fig. 3). In strain BZe327, exposure to disinfectant D resulted in increased reads associated with CRISPR sequences, ATP-binding cassette (ABC) transporter, ATP synthesis, and chemotaxis (**Table S8**). These gene classes are commonly associated with bacterial stress responses (Dawan and Ahn, 2022). Notably, ABC transporters frequently function as efflux pumps, contributing to AMR and virulence (Akhtar and Turner, 2022).

**Table 2:** Summary table of putative virulence DEGs in the treated strains with disinfectant F and D. Also included are the genes resulting from the comparison between the treated transcripts of the samples treated with antibiotics and disinfectants (ATB vs DIS).

	**Function**	**BZe322**		**BZe363**		**BZe327**
		**Disinfectant F**	**Antibiotics vs Disinfectants**	**Disinfectant F**	**Antibiotics vs Disinfectants**	**Disinfectant D**
*tonB*	Transport related genes	↑	↓	↑	↓	–
*tlyA*	16S/23S rRNA (cytidine-2′-O)-methyltransferase	-	↓	–	–	–
*shlB*	Type V secretion system	-	–	↑	–	–
*mexA*	Efflux pump component	↑	↓	↑	↓	–
*mexB*	RND transporter	–	–	–	–	↑
*phoP*	Two-component control system	–	↓	↑	↓	↑
*tetR*	Transcriptional regulator	–	↓	↑	↓	–
*mviN*	Cell wall biosynthesis	–	↓	–	↓	–
*mdtL*	Efflux pump component	–	↓	–	↓	–
*tolC*	Efflux pump component	↑	–	–	–	↓
*ylaC*	Transcriptional regulator	↑	–	↑	–	–
	Legend:	↑ = overexpressed	↓ = underexpressed	– = not differentially expressed		

### 3.6 Gene transcriptional analysis after the treatment with disinfectant based on sodium hypochlorite and caustic potash (F)

After the treatment of *A. butzleri* strains with F, many DEGs (log_2_ FC > 1 and < −1; *p-*value [FDR] < 0.05) associated with virulence and AMR were identified in strains BZe322 and BZe363 (Table 2, **Table S8**). A shared overexpression of TonB family protein was observed in both strains (log_2_ FC > 1.28 and log_2_ FC > 1.52, in BZe322 and BZe363, respectively) (Fig. 4, Table 2, **Table S9**). TonB is a cytoplasmic-periplasmic membrane protein. The overexpression of TonB-ExdB-ExdD complex has previously been reported under in vitro intestinal colonisation conditions (Buzzanca et al., 2023). This complex transduces energy from the inner membrane to the outer membrane transporters, allowing the uptake of substrates, such as iron-siderophore complexes. Its upregulation may represent an adaptive response facilitating bacterial colonisation and adhesion (Chiarini et al., 2024; Dykes et al., 2025) (**Table S9**). The exposure to disinfectant F in strain BZe322, induced overexpression of the multidrug resistance (MDR) gene *mexA* (log_2_ FC = 1.03). The *mexA* gene contributes to resistance to antibiotics and toxic compounds through its role in the MexAB–OprM efflux system, which mediates cellular detoxification (Chiarini et al., 2024). Upon the exposure to the sodium hypochlorite based disinfectant strain BZe363 overexpressed the virulence-associated gene *mviN* (Lipid II flippase MurJ) (log_2_ FC = 1.78 and log_2_ FC = 1.81, respectively, for Deseq2 and EdgeR analysis) (Table 2). This protein is involved in cell wall synthesis and has been implicated in resistance to antibiotics and toxic compounds (Buzzanca et al., 2023; Kumar et al., 2019). Furthermore, the DEGs involved in transcriptional regulation and methylation, *phoP* (a virulent gene expressed in two copies) (log_2_ FC = 2.49 and log_2_ FC = 1.32) and *rsmI* (log_2_ FC > 1.82), which is responsible for rRNA methylation and has been linked to resistance to disinfectants by modifying ribosomal structure (Lioy et al., 2014), were overexpressed after disinfectant exposure. Notably, two hypothetical co-regulated proteins were overexpressed in the strain BZe363, annotated as TolC family protein (log_2_ FC > 2.0) and TetR/AcR family transcriptional regulator (log_2_ FC > 2.26). The latter is involved in multiple cellular of processes, including detoxification and MDR function (Karatzas et al., 2008). The AcrAB-TolcC efflux system, a member of the Resistance-Nodulation-Division (RND) family, plays a key role in resistance to disinfectants and antimicrobial agents (Liu et al., 2025). Overexpression of the hemolysin transporter protein ShlB (log_2_ FC > 1.35), a component of the secretion system, in conjunction with TolC, was also observed. This system facilitates the secretion of toxins and other compounds (Reygaert, 2018).

Transcriptional regulatory DEGs, including *ylaC, tcrA,* and *czcR*, were up-regulated following exposure to disinfectant F in BZe322 and BZe363 (Fig. 4A; **Table S9**). In strain BZe322 was observed a high expression level of the RNA polymerase sigma factor *ylaC* (log_2_ FC > 4.44), co-regulated with the mobile element IS21 (log_2_ FC > 4.15) (**Table S8**). The *ylaC* gene was present two copies significantly up-regulated, highlighting its role as a key transcriptional regulator. YlaC is a pivotal component of the bacterial stress response, specifically involved in regulating genes associated with resistance to oxidative stress (Asai, 2017). The upregulation of IS21 and other mobile genetic elements may reflect stress-induced genomic plasticity, which has been previously associated with increased mobility and adaptive potential under environmental stress conditions (Baharoglu and Mazel, 2014).

Transcriptomic analysis of BZe363, following exposure to disinfectant F, revealed overexpression of *dksA* (log_2_ FC > 1.35) and tRNA pseudouridine synthase transcriptional regulator genes (log_2_ FC > 2.33) both located within the same genomic region. DksA has been shown to modulate ribosomal genes expression and stress-response pathways, while pseudouridine synthase contributes in translation protection. The coordinated expression of these genes suggests a coordinated multi-level response (Maharjan et al., 2023; Tagel et al., 2021), with pivotal roles of transcriptional adaptation and consequent protein synthesis. In the strain BZe363, the hypothetical proteins B_01651, B_01653 and B_01654 were annotated as a type II secretion protein (log_2_ FC > 3.77) (Korotkov, 2013), *zapB* (log_2_ FC > 1.53) which was involved in cell division (Galli and Gerdes, 2012) and *terB,* associated in tellurite resistance (log_2_ FC > 1.90). Consequently, this overexpression may indicate a potential adaptive or protective function. Notably, proteins involved in cell wall biogenesis were overexpressed in strain BZe363 as an adaptive response to disinfectant treatment, including lipopolysaccharide assembly protein B and outer membrane lipoprotein Blc (**Table S8**). Lipopolysaccharide assembly protein B plays a crucial role in membrane biogenesis and in bacterial survival (Klein et al., 2014). Its upregulation could be linked to a cell membrane repair response (Fivenson and Bernhardt, 2020). Blc protein is an outer membrane lipoprotein, expressed under stress conditions, suggesting a role in the adaptive response aimed at maintaining cell integrity (Sperandeo et al., 2008). Furthermore, GMP phosphodiesterase gene (log_2_ FC > 1.26) (**Table S8**), resulted in an overexpressed response to exposure to both disinfectants in strain BZe363. This protein is a pivotal regulator of biofilm formation, motility, and virulence and it’s a significant factor in tolerance under stress conditions (Lacey et al., 2010). MsrQ, a constituent implicated in oxidative stress (Gennaris et al., 2015), resulted overexpressed in BZe363 (log_2_ FC > 1.44) (**Table S9**). The function of MsrQ is to protect the cell damage caused by reactive oxygen species and chlorine, thereby ensuring its structural integrity (Gennaris et al., 2015).

Overall, the transcriptional profile induced by disinfectant F is consistent with a coordinated stress-adaptive response driven by oxidative stress. The simultaneous activation of genes involved in redox homeostasis, membrane remodelling, efflux systems, and global transcriptional regulation suggests an integrated regulatory network linking oxidative damage to bacterial survival strategies. In particular, the coupling between stress-response regulators and multidrug resistance determinants supports a model in which disinfectant-induced oxidative stress acts as an upstream signal promoting broad adaptive reprogramming rather than isolated gene-specific responses (Kohanski et al., 2010; Piddock, 2006).

### 3.7 Gene transcriptional analysis after the treatment with amine-based disinfectant (D)

Treatment of the *A. butzleri* strains with the amine-based disinfectant D led to the high expression of several virulence genes in strain BZe327 (Fig. 4B, 4D, Table 2). It is intriguing to note the expression of the efflux systems linked to antibiotic resistance, *mexB* (log_2_ FC > 2.27) and *arpC* (log_2_ FC > 2.12), which were co-regulated with genes involved in metal acquisition, the *fhuE* (log_2_ FC > 3.32) and *fecR* (log_2_ FC > 2.07) receptors. Notably *mexB* and *fhuE* were present in two copies (**Table S8**). MexB and ArpC, which were co-upregulated, are components of the MDR efflux system. This system is known to expel antibiotics, surfactants and disinfectants (Alsamhary, 2024; Chiarini et al., 2024). Under this exposure condition, genes associated with the ion transport of inorganic compounds and metabolism were differentially expressed. The *cntO* genes, present in multiple copies and belonging to this functional gene class, showed overexpression in BZe327 (log_2_ FC > 3.52) and were co-overexpressed with *mntP* (log_2_ FC > 2.37), *ccsA* (log_2_ FC > 4.06) and cytochrome C-552 (log_2_ FC > 1.11), genes linked to energy production and conversion (Jones and Joshi, 2021) (**Table S8**). MntP is involved in the transmembrane transport of manganese, an essential element for bacterial function, and enables defence against oxidative stress (Zeinert et al., 2018).

NikB protein, associated with nickel transport, and *cntO*, implicated in nickel and zinc transport, exhibited elevated expression levels in both BZe327 (log_2_ FC > 1.80) and BZe363 strains (log_2_ FC > 1.00) (Fig. 4B, **Table S8**), when exposed to the amine-based disinfectant (Maier and Benoit, 2019). This finding suggests a potential role in cellular detoxification, as indicated by its association with the transport of metabolites out of the cell. The co-expression of these genes may indicate of their involvement in the response to metal limitation or oxidative stress (Mulrooney and Hausinger, 2003).

In the strain BZe327, the *hypE* and *hypF* genes, which are linked to ion transport, were upregulated. *HypF* (log_2_ FC > 1.76) was co-regulated with hypothetical protein C_00970, whose function is unknown, but was strongly overexpressed (log_2_ FC > 4.26) as well as with Quinone reactive Ni/Fe Hydrogenase (C_00972, C_00973 and C_00974) (**Table S9**). HypF encodes a protein involved for hydrogenase maturation, an enzyme that catalyses the hydrogen redox reaction (Albareda et al., 2012). Specifically, HypE, together with HypF, is considered implicated in the addition of a C-terminal thiocarbamate group to HypE and in the formation of the CN ligand of the hydrogenase Ni-Fe metal centre (Albareda et al., 2012). This process is essential for hydrogenase catalytic activity and is conserved across diverse organisms (Albareda et al., 2012).

Furthermore, analysis revealed strong upregulation of genes encoding cell wall biogenesis, including *ftsA* (log_2_ FC > 2.33) and *ftsZ* (log_2_ FC > 4.57). These genes are hypothesised to participate in cell wall reconstruction, and were co-regulated with hypothetical proteins, including membrane solute transporters, nucleic acid metabolism and transcriptional regulators (**Table S8**) (Conti et al., 2018). Furthermore, in the case of strain BZe327, a higher percentage of DEGs related to CRISPR sequences was observed, particularly following exposure to disinfectant D. These genes including Cas1 and Cas2 endonucleases, were overexpressed (log_2_ FC > 2.00) co-regulated with CRISPR-associated protein Cas10/Cmr2 (**Table S8**). CRISPR-Cas complex is a defence mechanism against bacteriophages (Shabbir et al., 2019). In such cases, the defence mechanism was activated as a general response to stress, in a strain-specific manner.

### 3.8 DEGs following exposure to antimicrobials

Comparisons between general stress conditions (antibiotics and disinfectants) and control conditions didn’t reveal statistically significant differences related to gene expression (*p*-value > 0.05). However, DEGs were identified by comparing treated samples (**Figures S4, Tables S9, S10**). Strains BZe322 and BZe363, which shared several DEGs, including the virulence genes *murJ* (*mviN*), TonB family protein, and *phoP* (Fig. 4C, Table 2) (Chiarini et al., 2024). Simultaneous overexpression of these virulence genes increases the pathogenic potential of this microorganism (Buzzanca et al., 2021)*.* Disinfectants exhibit non-specific and multi-target mechanisms, affecting diverse physiological processes in a non-selective manner (Rozman et al., 2021).

**Fig. 5:**
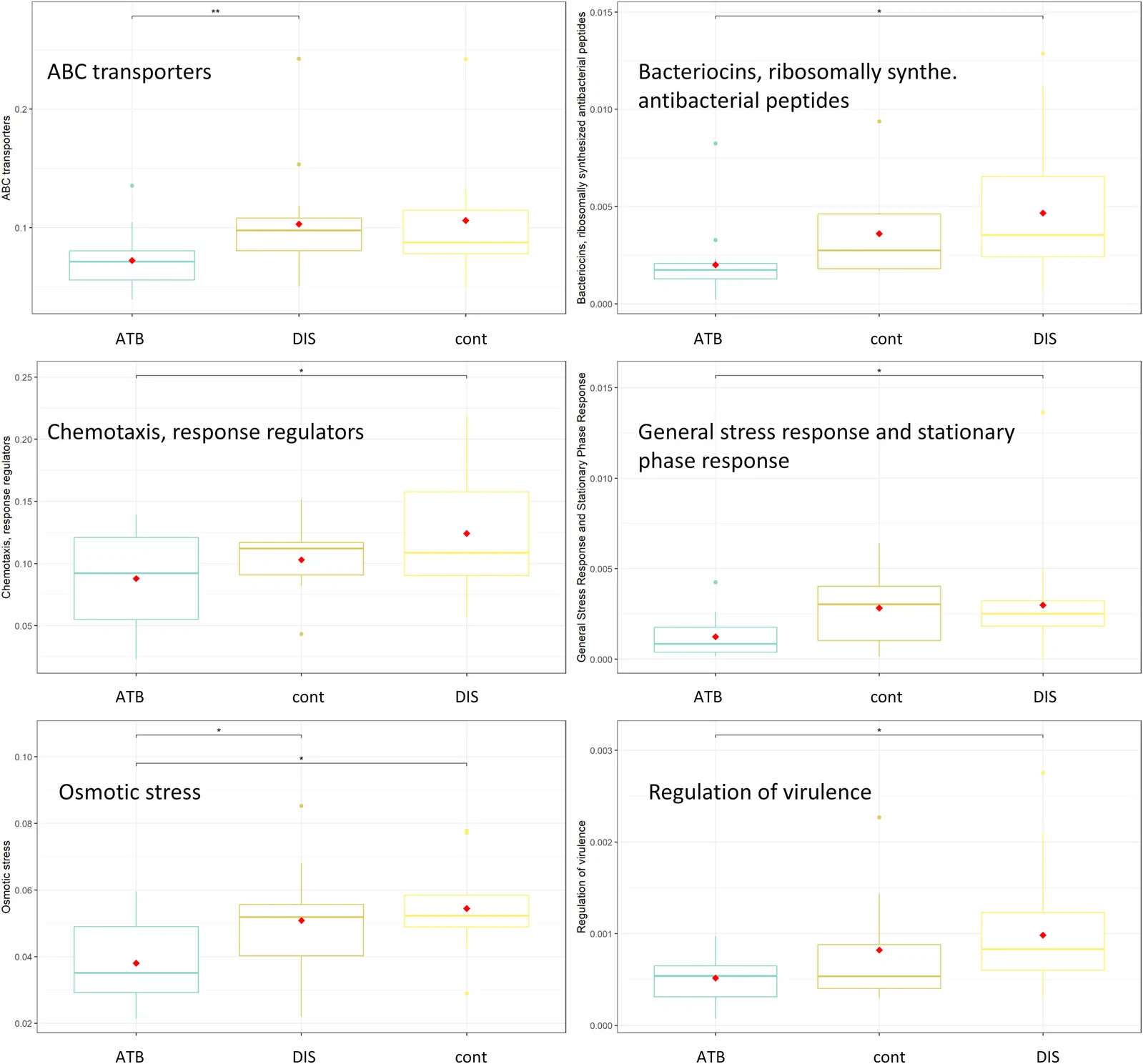
Percentage of reads related to the gene expression of *A. butzleri* under stress conditions (antibiotics = ATB, control = Cont, and disinfectants = DIS). The box-plots shown percentages of gene classes resulted differentially abundant between conditions. Asterisks indicate significance levels: **p-value < 0.01, *p-value < 0.05.

Functional analysis, considering the exposure conditions of *A. butzleri* strains, revealed upregulation of genes associated with ABC transporters, the production of bacteriocins, peptides, chemotaxis, osmotic stress and virulence regulation in response to disinfectant treatment compared with antibiotic exposure (Fig. 5, **Figure S5; Table S12**). Transporters and efflux systems represent fundamental mechanisms that facilitate the transportation of disinfectant compounds outside the cell membrane, providing a defence system for microorganisms (Li and Nikaido, 2009). Furthermore, chemotaxis, a functional class relevant to exposure to disinfectants, appears to be an additional fundamental defence system for antimicrobial resistance (Oliveira et al., 2022). The difference in exposure time between disinfectants (10–15 min) and antibiotics (60 min) reflects the distinct experimental setups and kinetic characteristics of these compounds. Disinfectants typically induce rapid, acute oxidative and membrane stress responses, whereas antibiotics such as erythromycin act through intracellular targets and may require longer exposure to elicit downstream transcriptional effects. Therefore, the observed transcriptional profiles should be interpreted in the context of these compound-specific dynamics rather than as a direct time-matched comparison.

## 4 Conclusion

This study analysed the resistance of selected *A. butzleri* strains to antibiotics and disinfectants using both in vitro and transcriptomic analyses to investigate the metabolic processes activated upon antimicrobial exposure. Studying antibiotic resistance remains crucial due to the high and continual adaptability of microorganisms to antimicrobial agents. Antibiotics and disinfectants trigger distinct response mechanisms. The limited number of DEGs observed after antibiotic exposure suggests restricted transcriptional remodelling despite the preserved metabolic viability detected by the MTT assay. This may reflect transient early adaptive responses not captured within the 60-minute exposure window or the induction of low-activity tolerance states resembling persister-like adaptations. In contrast, disinfectants induced a strain-specific response, depending on the type of product used, leading to the overexpression of previously described virulence genes in *A. butzleri* (e.g. *hecB, tonB, mexA, mexB, mviN*) (Buzzanca et al., 2021; Buzzanca et al., 2023), suggesting potential selection of virulence determinants following antimicrobial exposure. The results confirm the ability of these *A. butzleri* strains*,* previously isolated after sanitisation procedures (Botta et al., 2024), to resist disinfectant compounds by implementing strain-specific response mechanisms, including the overexpression of efflux systems, cellular detoxification systems, RNA modification and DNA recombination process. These resistance mechanisms may contribute to the ability of *A. butzleri* to survive also in the wild environment (Buzzanca et al., 2023). Therefore, careful manage of cleaning and sanitisation procedures in food processing environments, including the use of broad-spectrum products in rotation, is pivotal to counteract the diverse resistance strategies that microorganisms may develop. Further studies, including knock-out test are needed to evaluate the specific functions of genes that resulted in strong differential expression but were not identified in terms of function.

## Use of AI

Minor English editing was performed with the assistance of ChatGPT-4o (omni), developed by OpenAI (2025). After using this tool, the authors reviewed and edited the content as needed and take full responsibility for the content of the published article.

## Funding

This research was funded by Cassa di Risparmio di Torino (https://www.fondazionecrt.it/, accessed on September 4, 2025), grant number ALEV_CRT_20_01-Fondazione CRT 2019: Diffusion of *Arcobacter* spp. in Piedmont poultry meats and study of its pathogenic potential.

## CRediT authorship contribution statement

**Elisabetta Chiarini:** Writing – original draft, Visualization, Methodology, Investigation, Formal analysis, Data curation. **Davide Buzzanca:** Writing – original draft, Validation, Methodology, Investigation, Formal analysis, Data curation, Conceptualization. **Kurt Houf:** Conceptualization, Methodology, Supervision, Writing – review & editing. **Valentina Alessandria:** Writing – review & editing, Supervision, Resources, Project administration, Conceptualization, Funding acquisition, Methodology.

## Declaration of competing interest

The authors declare that they have no known competing financial interests or personal relationships that could have influenced the work reported in this paper.

## Data Availability

The RNA-seq raw sequence reads are available in the National Center for Biotechnology Information (NCBI) (https://www.ncbi.nlm.nih.gov/; link opened on 06/13/2025) under accession number PRJNA1248458.The raw sequences reads and related genomes of the 31 *A. butzleri* strains are available at the bioproject PRJNA986324. The functional annotations of the three *A. butzleri* selected strains are available on Zenodo at https://doi.org/10.5281/zenodo.18756660 (link opened on 02/24/2026).

## Appendix Supplementary materials

Image, application 1

Image, application 2
