# Supplementary material for: Resistance patterns and gene expression profiles of Arcobacter butzleri under exposure to selected antibiotics and disinfectants
Source: Curr Res Microb Sci. 2026 Jun 8;11:100631. doi: 10.1016/j.crmicr.2026.100631 (PMC13276354; doi:10.1016/j.crmicr.2026.100631)
Supplement: Supplementary file 1 [file mmc1.pdf]

Supplementary Figures

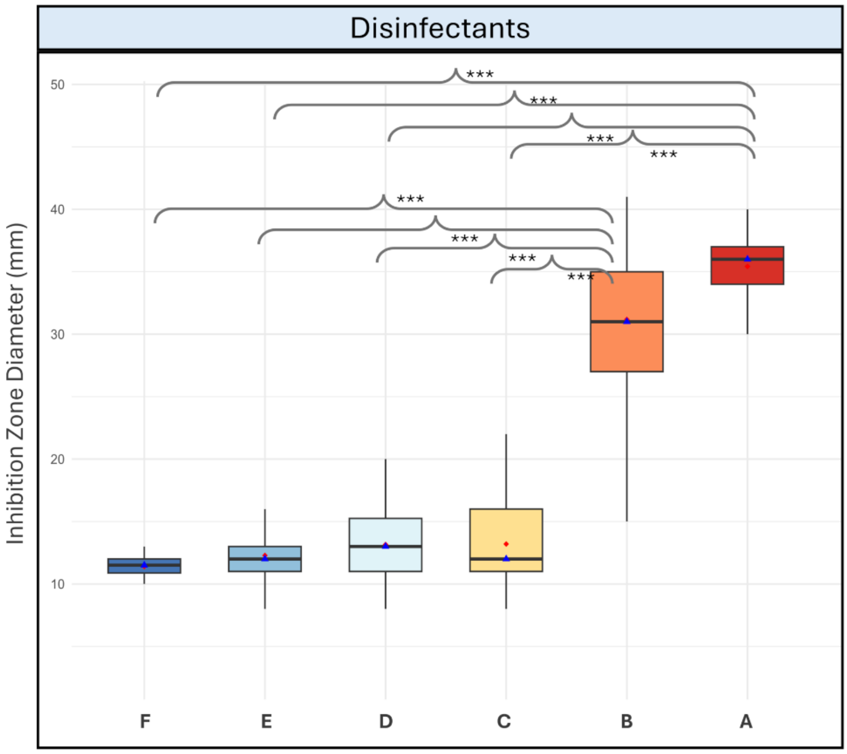

Figure S1. Boxplot showing the inhibition zone diameter (mm) produced by tested disinfectants (A–F). A blue triangle indicates the median, and the mean is marked by a red diamond. Statistical significance between groups was assessed using Kruskal–Wallis followed by Dunn's post hoc test (p-value < 0.05). Asterisks (***) indicate the significance level (p-value < 0.001).

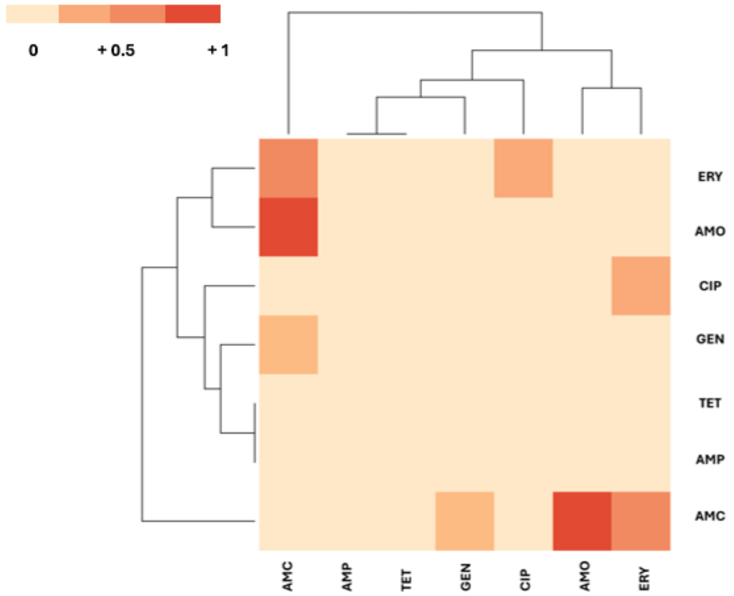

Figure S2. Binary Pearson's correlation matrix (0/1) showing co-resistance patterns between antibiotics; values represent the presence (1) or absence (0) of a resistance relationship. The number 1 was given to the resistant strain, while the number 0.5 was assigned to the intermediate resistance; finally, the number 0 was assigned to the susceptible phenotype. (**Table S5**).

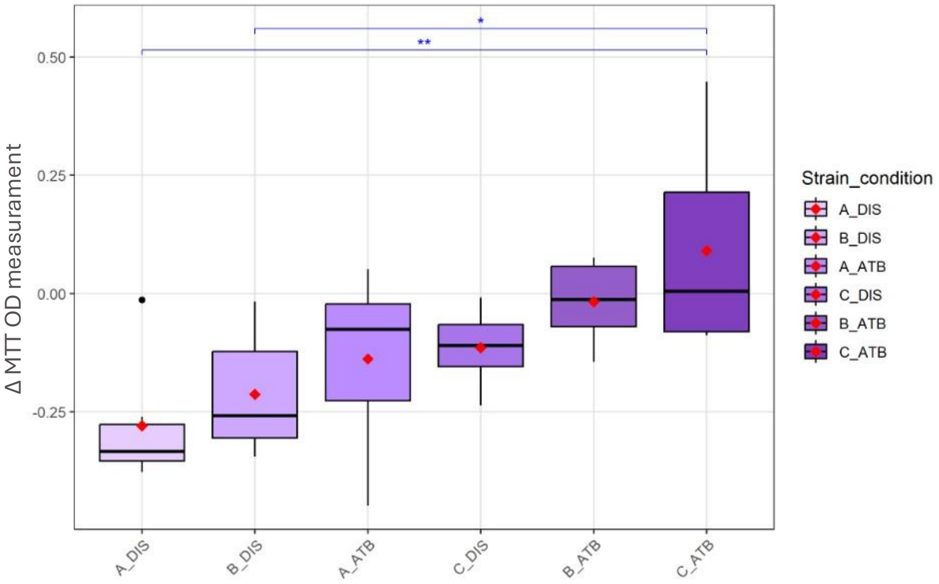

Figure S3. The box plots show the results of the OD ratio obtained following staining with MTT. The average results are represented by the red diamond. Strains A-BZe322, B-BZe363 and C-BZe327 were compared considering all antibiotics (ATB) and disinfectants (DIS) used in the treatment. Statistical significance between groups was assessed using the ANOVA/Tukey's test (* = *p-value* < 0.05; ** = *p*-value < *0.01*) (**Table S8**).

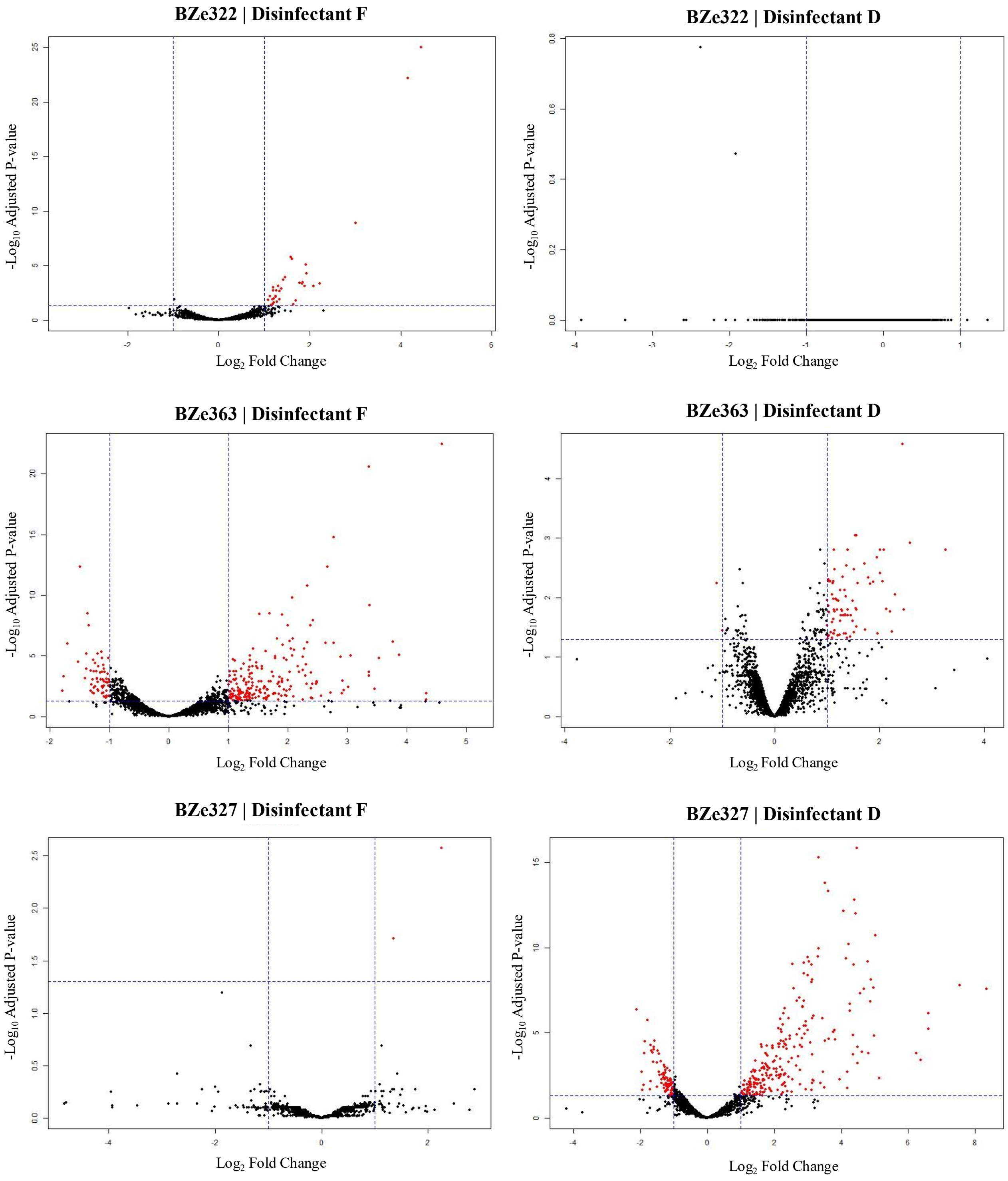

Figure S4. Volcano plots obtained from DESeq2 analysis for the three strains analysed (BZe322, BZe327 and BZe363) treated with disinfectants F (left panel) and D (right panel), respectively. The x-axis shows $\log_2$FC, while the y-axis represents the -Log_{10} of the corrected p-value (FDR). Red dots indicate DEGs ($|\log_2\text{FC}| > 1$, *p*-value [FDR] < 0.05), while black dots indicate non-significant genes.

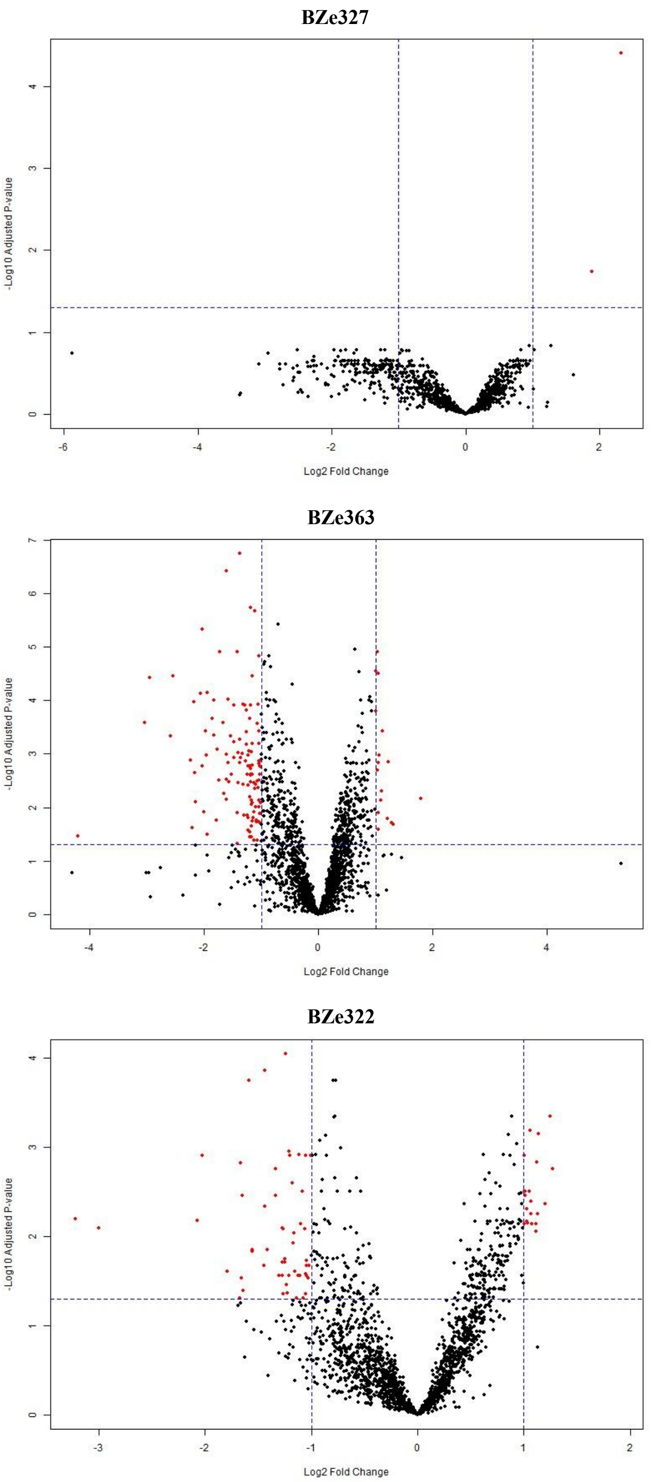

Figure S5. Volcano plots obtained from DESeq2 analysis for the three strains analysed (BZe322, BZe363 and BZe327) of the transcript obtained after the treatment with antibiotics compared to the transcript obtained after the treatment with disinfectant. The x-axis shows $\log_2$ FC, while the y-axis represents the $-\text{Log}_{10}$ of the corrected p-value (FDR). Red dots indicate DEGs (threshold: $|\log_2 FC| > 1$, *p*-value FDR < 0.05), while black dots indicate non-significant genes.
